# Assessment of Population Exposure to Coarse and Fine Particulate Matter in the Urban Areas of Chennai, India

**DOI:** 10.1155/2015/643714

**Published:** 2015-07-14

**Authors:** Ramachandran Prasannavenkatesh, Ramachandran Andimuthu, Palanivelu Kandasamy, Geetha Rajadurai, Divya Subash Kumar, Parthasarathy Radhapriya, Malini Ponnusamy

**Affiliations:** Center for Climate Change and Adaptation Research, College of Engineering, Anna University, Guindy Campus, Chennai, Tamil Nadu 600 025, India

## Abstract

Research outcomes from the epidemiological studies have found that the course (PM_10_) and the fine particulate matter (PM_2.5_) are mainly responsible for various respiratory health effects for humans. The population-weighted exposure assessment is used as a vital decision-making tool to analyze the vulnerable areas where the population is exposed to critical concentrations of pollutants. Systemic sampling was carried out at strategic locations of Chennai to estimate the various concentration levels of particulate pollution during November 2013–January 2014. The concentration of the pollutants was classified based on the World Health Organization interim target (IT) guidelines. Using geospatial information systems the pollution and the high-resolution population data were interpolated to study the extent of the pollutants at the urban scale. The results show that approximately 28% of the population resides in vulnerable locations where the coarse particulate matter exceeds the prescribed standards. Alarmingly, the results of the analysis of fine particulates show that about 94% of the inhabitants live in critical areas where the concentration of the fine particulates exceeds the IT guidelines. Results based on human exposure analysis show the vulnerability is more towards the zones which are surrounded by prominent sources of pollution.

## 1. Introduction

There is an increased concern among policy makers regarding the escalation of particulate pollutants in urban cities around the world. The coarse particulate matter (PM_10_: particles with aerodynamic diameter 2.5–10 *μ*m) and the fine PM (PM_2.5_: particles with aerodynamic diameter lesser than 2.5 *μ*m) are hazardous to climate and health [1]. These particulates in outdoor air pollution were recently designated as Group I carcinogen by the International Agency for Research on Cancer [2]. Many epidemiological studies have shown that particulates, especially inhalable particulates, are harmful to human health. Therefore, particulates have been selected as indicators in the evaluation of air pollution that damages health, as per the World Health Organization (WHO) [3–6]. It is estimated that 2.6–4.4 million premature deaths per year are reported due to poor air quality (AQ) conditions [7]. Numerous epidemiological studies indicate that both long- and short-term exposure to atmospheric PM are associated with increases in mortality and morbidity [8–13]. In general, most of the studies represent only the impact caused by the pollutant in various time scales. It is indeed important to assess the population exposure to various levels of the pollutant in the spatial and the temporal scales [14–17]. Air pollution exposure assessment studies are limited in developing countries like India, due to their widespread geographical coverage, increased urban population sprawl, and limited number of air pollution monitoring stations. These gaps can be reduced by implementing various geospatial modeling approaches. Relevant studies have already been reported for China on its various climatic regions and cities such as Lanzhou, Beijing, and Mainland China using geospatial approaches [3, 4, 6, 8, 17, 18]. Poor AQ in Indian cities continues to present an ominous picture for health burdens attributable to ambient air pollution. Analysis of routinely collected ambient AQ data released by the Central Pollution Control Board (CPCB), Government of India, indicates that the annual average concentrations of PM_10_ are critically high defined as >90 *μ*g/m^3^ by the CPCB based on more than half of the 503 locations monitored across the country [19]. Furthermore CPCB has classified >90 *μ*g/m^3^ as a critical cutoff value for the industrial, residential, rural, and other locations [20, 21].

Studies that measure health impacts and population exposure to pollution are effective in increasing the awareness about AQ in India and serve as a call for action. Various reports from the CPCB claim that the AQ in Chennai is moderately polluted when compared with other Indian cities [20, 22]. Chennai is located on the northeast end of Tamil Nadu on the seashore, off the Bay of Bengal. It lies between 12°9′ and 13°9′ N latitude and 80°12′ and 80°19′ E longitude. It is the fourth largest metropolitan city in India. According to the latest population census, the city had 8.68 million residents, making it the sixth most populous city in India (Department of Census, Government of India 2011). The climate of Chennai is tropical, wet, and dry. Since it lies along the coast, there is much less seasonal variation in the temperature. The average highest temperature is 33.3°C and the average lowest temperature is 23.8°C. The average annual rainfall is approximately 140 cm and the city gets most of its seasonal rainfall from the northeast monsoon winds from the mid-October to mid-December months. With its proximity to the Bay of Bengal and thus access to markets in East Asia, Chennai is an important busy port city. Besides trade and shipping, the automobile industry, software services, medical care, and manufacturing industry form the foundation of the economic base in Chennai. The city has an expanded urban sprawl of 1195.02 km^2^; however, only six air pollution monitoring stations are operated under the National Ambient Air Quality Monitoring Programme (NAAQMP), which monitors gaseous and coarse pollutants weekly twice manually. In addition, two Continuous Ambient Air Quality Monitoring Stations are operated by NAAQMP. These monitors are not located at the prime pollution source points such as dump yards and brick kilns to depict the real status of the AQ of the city. A recent study from the Berkeley University shows that Chennai ranks in the fourth position in terms of grams of pollution inhaled per gram of pollution emitted among the mega cities in India [23, 24]. This present study focuses on the evaluation of human exposure to coarse and fine PM of Chennai using population-weighted exposure level (PWEL) algorithm. Geospatial statistical methods were used to analyze the spatial extent of the pollution and to evaluate the vulnerability of the population to particulate pollution based on WHO AQ guidelines (AQGs). The results can serve as a useful reference for health risk management, and they can serve as a vital decision-making tool to concentrate on sources that need more attention in order to curb the pollutant burden.

## 2. Materials and Methods

### 2.1. Site Description

**Figure 1:**
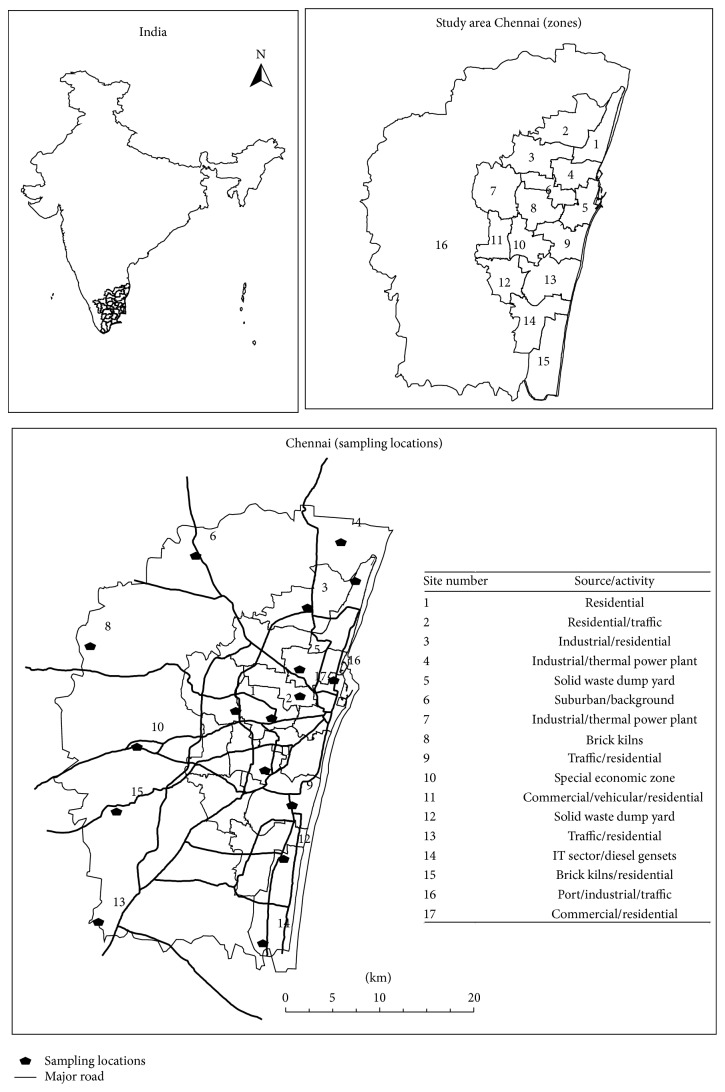
Study area.

PM_10_ and PM_2.5_ data were collected by systematic sampling from 17 source-based locations of air pollution in Chennai (Figure 1). The samples were collected between 1 November 2014 and 31 January 2015. The sampling sites were split into 4 zones, and 12 samples consisting of 24-hour average concentration were collected from each location during the study period. Standardized sampling procedure, including selection of sampling sites, instrumental checks, and handling of samples, was followed as per the guidelines provided by the CPCB ambient AQGs. Owing to equipment and power failure, we have limited ourselves to small sampling size from each location. In addition, meteorological parameters such as wind speed and wind direction were obtained from the ECMWF ERA data (at 6 h interval). There were few precipitation events during the first and the third week of November, during which period limited sample was collected from the study area.

### 2.2. Population Data for Chennai

Population count gridded data derived from the Center for International Earth Science Information Network contains the human population distribution. It is the gridded data product that renders global population data on the scale and extent required to demonstrate the spatial relationship of human population and the environment across the globe. Gridded population of the World version 3 has been extensively used in various global population studies [25, 26].

### 2.3. Spatial Interpolation of Particulate Pollution Concentration

In the present study, the particulate matter monitoring sites were selected after a detailed survey of air pollution sources in the city. Emission sources like industrial clusters, petrochemical refineries, and power plants were selected from point emitting sources. Diffused sources such as the vehicle exhaust, open waste burning, and road dust were also included in the study. Figure 1 represents the study area map with location of air pollution sources in Chennai. Activity data and source information were collected from the existing air pollution inventory for the Chennai city reported by Guttikunda et al., (2012, 2014) [24, 27]. Although the 17 sampling locations were selected to represent the whole spatial extent of Chennai, we have used interpolation technique in Arc GIS 9.3 platform to surface data over the entire area. Since there are numerous spatial interpolation methods, this study used the Kriging method, originated by Matheron 1963 [28]. This method is one of the popular methods used in various environmental research applications [4, 29, 30].

### 2.4. Calculation of Population Exposure to PM Based on AQGs

The PM data (PM_10,2.5_) from the study period were averaged for all the sampling locations and interpolated at the resolution of 0.01 × 0.01, approximately 1 km × 1 km. Gridded population data were converted to points and spatially interpolated, to attain the high spatial resolution (0.01° × 0.01°) matching that of PM concentration data.

Using the GIS, the interpolated pollution data were classified into four classes according to the WHO 24-hour mean level interim target (IT) guidelines for PM_10,2.5_ as given in Table 1. The pollution layers pertaining to each class were converted into polygon features, which were used to analyze the population exposure levels and total vulnerable area under various concentration ranges categorized.

### 2.5. Calculation of Population-Weighted Exposure Level

In this study, population-weighted exposure level (PWEL) was calculated in the 15 administrative zones and suburban area of the revised Chennai corporation collectively comprising a total area of 1195 km^2^ known as the Chennai metropolitan area. To obtain the total population exposure under the AQGs, the population density of the each zone was calculated using

(1) $$\begin{array}{c} \text{Population density},\;\;d_{i}=\frac{p_{i}}{A_{i}}, \end{array}$$

where *d*_*i*_ denotes the geographical unit population density of the zone, *p*_*i*_ the population unit of the zone *i*, and *A*_*i*_ its area unit of the zone *i*.

The PWEL of the given grid *i* is calculated based on the exposure equation as follows:

(2) $$\begin{array}{c} \text{PWEL}=\frac{\sum \left(P_{i}\cdot C_{i}\right)}{\sum P_{i}}, \end{array}$$

where *P*_*i*_ is the population in the grid *I* and *C*_*i*_ is its average PM_10,2.5_ concentration.

## 3. Results and Discussion

### 3.1. Particulate Mass Concentration

**Figure 2:**
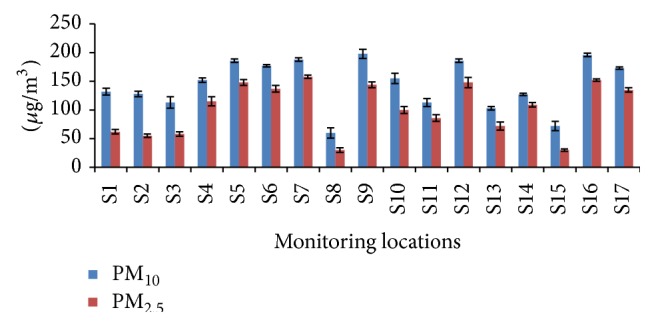
Average PM_10,2.5_ concentrations for sampling locations in Chennai.

Figure 2 represents the mean coarse and fine particulate concentration of the sampling points during the study period. The concentrations of the coarse and the fine particulate ranged from 60 to 198 *μ*g/m^3^ in coarse mode, whereas the latter showed concentrations from 30 to 158 *μ*g/m^3^. In recent years there has been a drastic increase in the total number of motor vehicles in Chennai and there has been a 57-fold increase in the vehicle number since 1975 [31]. The summary of the pollution sources in Chennai is presented in Table 2. A recent study by Harvard University used ground based particulate data from CPCB along with satellite observation reveals that over half of India's population live in areas that exceed the Indian National Ambient Air Quality Standard for fine particulate pollution. They predicted that the life expectancy of the people living in the vulnerable area will be reduced to 3.2 years in the total lifetime [32].

### 3.2. Population Exposure Based on AQGs

Table 3 represents the total geographical area, demographic details, and the population densities of each zone. It clearly indicates that zone 5 has the highest population density, limited to 1.78% of the total area. We observed that the population density of the others zones (4–6, 9, and 10) such as the northern and the central part of Chennai was also generally higher. Consecutively, zones in the southern and the western regions (8 and 13) were also comparatively densely populated. In general, the population density of the city ranges from 2190 to 23,106. Ultimately, this indicates that all the zones are influenced by the demographic factor, and this will represent an additional feedback in exposure weighting.

**Figure 3:**
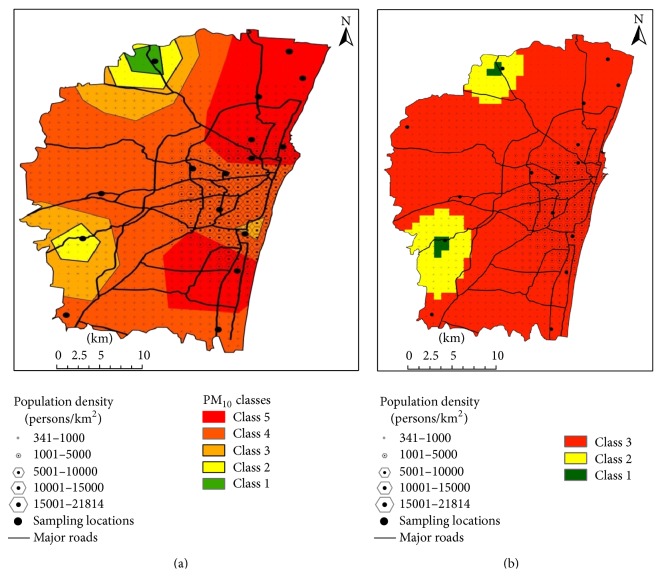
(a) PM_10_ vulnerability map of Chennai. (b) PM_2.5_ vulnerability map of Chennai.

Figures 3(a) and 3(b) represent the spatial distribution of the PM_10,2.5_ pollution in the study area, which clearly reveals that the high concentration levels of the pollutants were more centered towards the high pollution sources.

Table 4 reveals the percentage of area and the population categorized under the PM_10_ AQGs. Among them, class 1 represents the area that exceeds the PM_10_ AQGs, that is, above 150 *μ*g/m^3^. Even though, this class comprised only 24.51% of the total area; it covers a population of 28.09% of the total population. These regions are also highly vulnerable to continuous exposure to particulate pollution. It is to be noted that the majority of the population in the study domain lives in class 2 of the vulnerability class where the concentration of the pollutants ranges from 150 to 100 *μ*g/m^3^. This class holds a total population of 68% comprising 61% of the total geographical coverage. Interestingly, it also has a population density of 5352 persons/km^2^ and this shows that most people are exposed under this category. It is to be noted that only small portion of the population, that is, 4%, <1% of the people, live in classes 3 and 4 of the vulnerability category.

**Figure 4:**
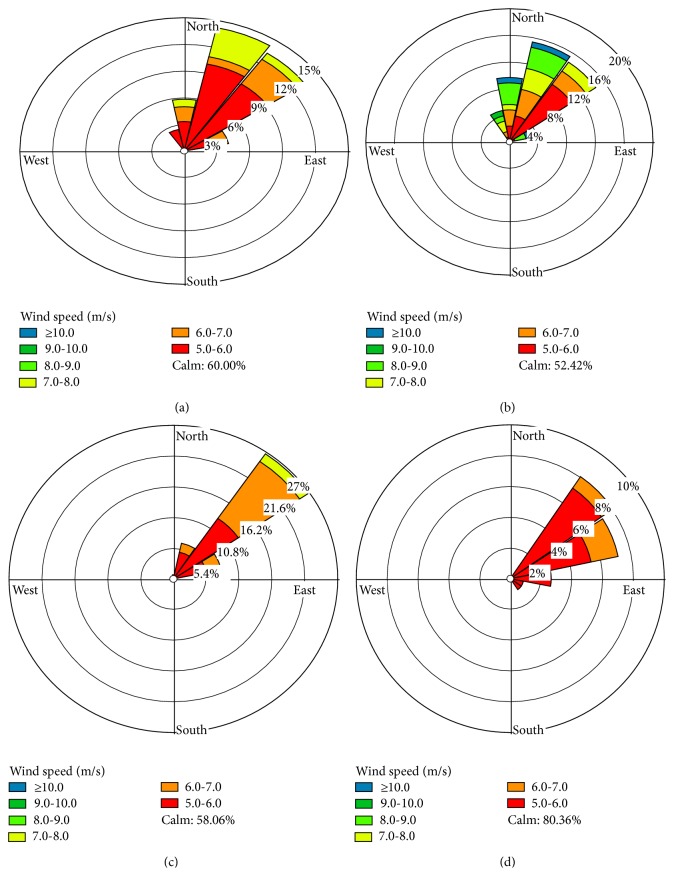
Monthly wind speed (m/s) and wind directions over Chennai. Data derived from ECMWF (at 6-h interval), (a) November 2013, (b) December 2013, (c) January 2014, and (d) February 2014.

As suggested in the IT guidelines, there will be about 2.5–5% increase of short-term mortality in regions where the PM_10_ target level exceeds 150 *μ*g/m^3^. However, the Indian ambient AQ standard for the PM_10_ is fixed as 100 *μ*g/m^3^ for 24-hour period. Clearly, these results indicate that the majority of the vulnerability classes in the city reside in the probable risk zones which can induce short-term mortality. It is clearly evident from the previous studies conducted in the USA and China for population exposure assessment for PM_10_ which report that more people are residing in the localities that are prone to higher concentrations of coarse pollutants [3, 11, 15, 18]. The results from the PM_2.5_ exposure classes are furnished in Table 5. When compared with the PM_10_ exposure levels, an alarming 94.34% of the total population, which comprises 88.57% of the total geographical area, is categorized under high exposure-sensitivity groups exceeding above the PM_2.5_ AQ IT guidelines. This shows that a majority of the city is exposed to PM_2.5_ concentrations above 75 *μ*g/m^3^. However, the Indian AQGs for PM_2.5_ are fixed as 60 *μ*g/m^3^ for 24-hour period. The results show that the majority of the population resides in locations where they are prone to high risk of health impacts induced by fine PM. It is important to note that only a small proportion (0.29%) of the population is classified under PM_2.5_ range of 50–37.5 *μ*g/m^3^. Incredibly, there is no population group which is set to be at the safe limits of PM_2.5_ concentration below 37.5 *μ*g/m^3^. A summary of monthly wind speed and wind direction is represented in Figure 4. It shows that, during the study period, the wind direction shows that majority of the wind flows from northeast (NE).

The interplay between the land and sea breezes has a significant factor for scavenging the pollution from the industrial clusters, petrochemical refineries, and the power plants, getting dispersed to the sea and thus reducing their impact on the urban air quality. The same cannot be considered for the diffused sources in the city, such as the vehicle exhaust, open waste burning, and road dust. [27], Wind circulation may lead to more exposure to the pollutants in other interior locations as well. Already the city is facing a unique problem such as the 6th urban areas population projections states that Chennai will attain 11 million population by 2030 [37]. As a result population and the vehicles in the city will further add to the burden of the particulate pollution as well as congestion [38]. The population density at the center of the city is considerably higher, when compared with other regions. The figure shows that the prevailing meterological conditions will favor the dispersion of the pollutant load from the northern part being transported to the inner core of the city during the study period.

The northern region harbors major pollution sources like thermal power plants, oil refineries, brick kilns, open dump site, and commercial port. Furthermore, industries are located on the western side of the city along with the cluster of brick kilns. Since the city is well known for industrial, commercial, and port activity, it attracts lots of heavy vehicles for freight movement. The city operates most of the heavy vehicular movement through national highways from the outer city during the day hours, while in the night hours the heavy vehicles are allowed to use the city road network that pass by heavily congested population zones. This leads to increase in pollutant from the diesel trucks making people more exposed to particulate matter. In densely populated city like Chennai, more than 50–60 percent of the population live or work near the roadside where levels are much higher. This is very serious in low income neighborhoods located close to roads. Road users, public transport users, walkers, and cyclists are the most exposed groups and they are also the urban majority.

Chennai is considered as the second largest IT hub next to Bangalore; most of the corporate companies and shopping malls in the city use diesel sets to run their air conditioner systems. Particularly during summer the demand for electricity increases; it drives the increased usage of diesel generator sets to meet out the demand. Regulatory agencies should ensure that the diesel engine is checked for every six months to comply with the emission standard. The city has seen a sudden increase of construction sector and allied industries in the past decade; around 600 traditional bricks manufacturing industries are surrounded in the west of the Metropolitan city. The fixed chimney in the bull trench kilns were designed with stacks of at least 50 m, which allow for the emissions to travel farther than the low lying sources [24]. Moreover it increases the diesel trucks in the city for movement of bricks to the concerned locations.

Table 6 represents the research studies conducted on source apportionment studies in Chennai, providing the affirmative information that coarse pollution arises from the sources in the order of transport, resuspended road dust, industries, and power plants. However, the fine pollutant emitted majority from transport, power plants, industries, and brick kilns.

The methodology used in the study will provide the knowledge about the spatial spread of the pollution. Furthermore, this method provides the information about the population residing in pollution hotspots. The additional advantage of using the method is with limited information about the pollutant; it can be used as an effective tool for decision support system. The results from the exposure, sensitivity studies reveal that the assessments will be further enhanced when it is coupled with the land use and pollution dispersion modeling [17, 24, 36, 39–42].

### 3.3. Calculation of PWEL Population-Weighted Exposure Level Concentration

Overall, human exposure to PM_10,2.5_ calculated with population weighting is slightly higher than the mean concentrations in most of the regions in the study area. In general, the spatial distribution of the population settlement grows from north and is more concentrated on the central and the southern zones of the city. The distribution of the population density is one of the most important factors considered for the calculation of the population exposure to particulate pollution. It is independent of the total geographical area or the total population covered in the zone. While calculating PWEL for each zone it has to be considered that the exposure increases with increasing population density.

**Figure 5:**
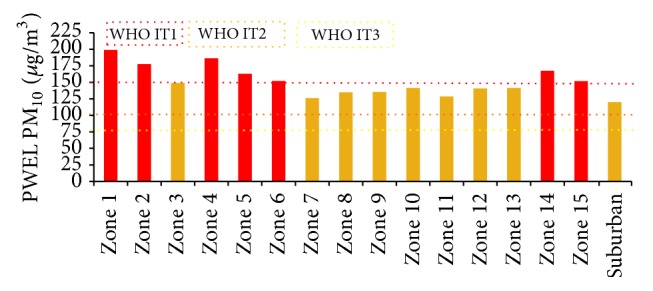
PM_10_ PWEL of zones in Chennai under WHO IT guidelines.

**Figure 6:**
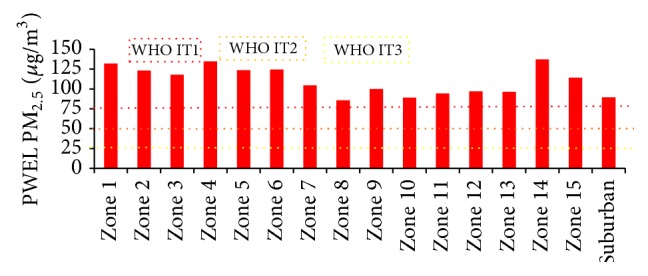
PM_2.5_ PWEL of zones in Chennai under WHO IT guidelines.

A similar trend of results was observed for the PWEL for both the particulate pollutions represented in Table 7. The regions with higher population densities such as Tondiarpet (Zone 4), Royapuram (Zone 5), Thiruvika Nagar (Zone 6), Anna Nagar (Zone 8), Teynampet (Zone 9), Kodambakkam (Zone 10), and Adyar (Zone 13) show a prominent increase in exposure levels when weighted with demographic factors. Similar results were observed for coarse pollutants in various regions in China. They depict that more population weighting is concentrated towards the densely populated zones which display high risk owing to particulate pollution [3, 17, 43, 44]. The results from Figures 5 and 6 represent the individual weightage of the zones after exposure analysis, and it is classified as per the IT guidelines for both the pollutants. These results clearly reveal that the sensitivity of the person exposed to the coarse pollutants (Figure 5) is more in Zones 1, 2, 4–6, 14, and 15, which exceeds the IT guidelines. Even though the zones such as 1, 2, 3, 7, 14, and 15 have moderate population density, these zones are surrounded by thermal power plants and petrochemical industries and technological parks. Hence, due to the presence of high pollution concentration, the population residing at these zones should be treated as a high-vulnerable class. More explicitly, Zones 3, 10, 12, and 13 are in the brink of Class I guidelines that even with a small increment either in the population or the pollution level they will exceed the AQGs for coarse pollutant. However, the PWEL for PM_2.5_ (Figure 6) shows that the human exposure to the fine particulate pollution is considerably severe at all zones where the exposure level exceeded the AQ IT class I guidelines of 75 *μ*g/m^3^. This shows that the PWEL for PM_2.5_ ranged from 85 to 136 *μ*g/m^3^. These indicate that the populations residing in the study area are exposed to higher concentrations of coarse particulate pollution. A study based on the health impacts of particulate pollution in Delhi demonstrated similar results that the average concentrations of the pollutant were extremely higher than the national AQ norms at all the regions [45, 46].

## 4. Strategies to Control Particulate Pollution in Chennai

The study conducted by the CPCB (2010 [35] for the source apportionment indicated that dust on paved roads has the bigger share among particulate matter when compared to that of vehicular emission. The panel suggested that roads be swept and watered to bring down the amount of road dust. The Chennai Corporation has already taken initiative to reduce the proposition of road dust by replacing manual sweepers by mechanical seeping systems as a trial basis in selected zones of corporation. Similarly it has been shown that the vehicle registration has shown 57-fold increase from 1975; among them cars contribute 20% and two-wheeler shares 55 and of the total vehicular fleet, other than this since it is port based city it attracts more numbers of diesel based trucks for freight management. Currently most of the vehicles in Chennai ply on petrol, diesel, and a small proportion of LPG with Bharat stages 3 and 4 standard fuel. Government should frame a strategy to increase the number of buses and trips in peak hours in strategic locations, further after effective operationalization of the ongoing metro rail service will bring down the vehicle contribution.

A recent study conducted by a civic society reveals that proper maintenance of the city buses and replacing old buses with new ones will reduce the pollution burden from the public carrier sector. It is the responsibility of the policy makers to take initiatives by providing the LPG and CNG in the subsidized rate to encourage the public usage. However, the city commuters are reluctant to opt cleaner fuels, due to the insufficient supply of the LPG and CNG. In a recent development that Chennai Corporation identified 71 bus route roads to extend the pedestrian footpaths, cyclist pathways to enhance the walk ability to ease the congestion. Focus should also be on improved design for shared and common public spaces. Experience from around the world shows that parking controls, parking pricing along with taxes top the list as the first generation car restraint measures worldwide.

The city corporations have decided for scientific closure of the existing two open dump yards to whip off the menace from open burning. It is in the process of finalizing the methodology for effective waste management. To meet out the pollution contribution from large share of industries and power plants in the city, government should implement technologies like flue gas desulfurization and carbon sequestration will bring down the formation of secondary sulfate pollutant from those sectors. It is proven that the emerging technologies in the brick kiln sector such as vertical shaft brick kilns (VSBK), zigzag, and Hoffmann could result in at least 40% reduction in the PM emission rates alternative to currently in-use clamp and bull trench [47].

## 5. Conclusions

The results reveal that all the zones have a positive impact to the particulate pollution after the human exposure weighting. Zones which are surrounded by dense pollution and economic activities show a considerable increment in the weighting analysis. Alarmingly, the results from the exposure assessment indicate that the majority of the people live in the risk zones. This research outcome demonstrates that the particulate pollution situation is not affirmative in the study area. There is a wider gap in fulfilling both the particulate concentration and WHO guidelines in view of health effects caused by PM.

Particularly the results from the fine pollutant reveal that alarmingly 95% of the inhabitants in the city live in class 1 vulnerable zone of IT guidelines. This strongly warrants the importance of reducing the concentration levels of particulate pollutants by framing strict management policies and regulatory systems. This could be achieved by periodical monitoring and enhancing stringent supervision by the environmental authorities.

## Figures and Tables

**Table 1:** WHO IT guidelines for coarse and fine PM.

24-h mean level	PM_10_ (*µ*g/m^3^)	PM_2.5_ (*µ*g/m^3^)	Basis for the selected level
IT-1	150	75	About 5% increase of short-term mortality over AQG
IT-2	100	50	About 2.5% increase of short-term mortality over AQG
IT-3	75	37.5	About 1.2% increase in short-term mortality over AQG
AQG	50	25	Based on relation between 24-hour and annual PM levels

**Table 2:** Summary of pollution sources in Chennai.

Sources	Numbers
Vehicle fleet (millions)	4.2 (2013)
Cars and jeeps	682434
Two wheelers	3339933
Three wheelers	74619
Buses + stage carriers	48672
HDV + LDV + others	145,865
Coal-fired power plants	3
Brick kilns number and type	600, bull trench
Other prominent sources	Re suspended road dust, open dump sites burning, diesel gene sets, construction activities, and so forth

Some of the information adopted from Guttikunda et al. 2014 [27].

**Table 3:** Demographic and area proportion of zones of Chennai.

Zone name & number	Population ratio (%)	Area ratio (%)	Population/ area
(1) Thiruvotriyur	1.76	2.07	4101
(2) Manali	1.51	3.31	2190
(3) Madhavaram	2.72	2.79	4688
(4) Tondiarpet	4.77	1.77	12,963
(5) Royapuram	8.54	1.78	23,106
(6) Thiru. Vi. Ka. Nagar	3.62	1.42	12,255
(7) Ambattur	3.44	3.19	5187
(8) Anna Nagar	6.78	2.11	15,443
(9) Teynampet	8.74	2.14	19,717
(10) Kodambakkam	5.26	1.88	13,490
(11) Valasaravakkam	2.41	1.70	6832
(12) Alandur	3.31	2.16	7371
(13) Adyar	8.37	3.35	12,058
(14) Perungudi	4.50	2.99	7253
(15) Sholinganallur	2.88	3.52	3944
Suburban	31.39	63.73	2379

**Table 4:** PM_10_ vulnerability classes.

Class	PM_10_ pollution range (*µ*g/m^3^)	Population ratio (%)	Area ratio (%)
1	>150	28	25
2	100–150	68	61
3	75–100	4	12
4	<75	<1	2

**Table 5:** PM_2.5_ vulnerability classes.

Class	PM_2.5_ pollution range (*µ*g/m^3^)	Population (%)	Area ratio (%)
1	>75	94	89
2	75–50	5	10
3	50–37.5	1	1
4	<37.5	0	—

**Table 6:** Previously reported source apportionment studies for Chennai city.

SI. number	Source/locations	Author & year	Contribution
1	Mixed/traffic, industrial, background sites	Oanh et al., 2006 [33]	Traffic, industrial showed dominant source signatures. Ratio between PM_2.5 _and PM_10_ was about 0.5 at all the three sites during 2002-2003

2	Industry & road dust	Central Pollution Control Board (CPCB), 2010 [34]	PM_10_ & PM_2.5_ showed the highest levels of traffic and industrial site during winter (December–February 2001-2002). Source segregation provided dominance by resuspended soil/road dust

3	Industry, traffic, background & kerbside	Bathmanabhan and Madanayak, 2010 [35]	Results from the factor analysis showed that diesel based engine (DG sets and vehicles), gasoline vehicles, and soil dust are major sources of pollution in most of the sites and in most of the season

4	Road side/traffic location	Ragettli et al., 2014 [36]	Relationship between PM_2.5_ and PM_10_ concentrations showed a significant correlation denoting that traffic related emissions are the main source contributor at the study location

5	Multipollutant gridded emission inventory & dispersion modeling from all available sources like transport, road dust, residential, power plants, industries, brick kilns, waste burning, and diesel generator sets	Guttikunda and Jawahar, 2012 [24]	As PM_10_ concern road dust contributes as the prime source with 41%, followed by transport (20%), power plants (11%), brick kilns (7%), construction activities (6%), domestic and waste burning (5%), industries, and diesel generator sets share 2%, whereas PM_2.5_ relates to transport (35%) followed by power plants (14%), road dust (12%), brick kilns (11%), domestic & waste burning shares (8%), diesel generator sets, industries, and construction activities share (4%)

6	Sector-specific emissions inventory from all known sources	Guttikunda et al., 2014 [27]	Estimated particulate emissions inventory for the base year 2012 shows that for PM_10 _major contributors are listed as transport sector (34%), industries (21%), power plants (12%), road dust (9%), brick kilns (7%), domestic & construction (4%), open waste burning (3%), generator sets (1%). Similarly, contributions from PM_2.5_ show industries (21%), power plants (13%), brick kilns (7%), domestic (6%), open waste burning (3%), road dust (2%), and generator sets & construction (1%)

**Table 7:** PWELs for each zone in Chennai.

Zone	Average PM_10_	PWEL PM_10_	Average PM_2.5_	PWEL PM_2.5_
Zone 1	198.17	198.94	131.20	131.88
Zone 2	177.17	177.51	122.62	122.96
Zone 3	148.00	148.88	116.78	117.72
Zone 4	185.21	186.39	133.18	134.57
Zone 5	159.48	162.98	121.05	123.48
Zone 6	150.97	152.05	123.24	124.45
Zone 7	125.47	125.90	103.75	104.63
Zone 8	133.96	135.02	84.41	85.72
Zone 9	133.46	135.46	98.02	99.89
Zone 10	140.51	141.60	87.90	89.06
Zone 11	127.84	128.74	93.56	94.22
Zone 12	140.43	140.52	96.15	96.91
Zone 13	140.07	141.52	95.23	96.37
Zone 14	166.43	167.51	136.33	137.02
Zone 15	151.23	151.95	113.78	114.11
Suburban	119.21	119.75	89.21	89.51
